# Pedigree-based genetic dissection of quantitative loci for seed quality and yield characters in improved soybean

**DOI:** 10.1007/s11032-021-01211-6

**Published:** 2021-02-06

**Authors:** Wenxuan Huang, Jingjing Hou, Quan Hu, Jie An, Yanwei Zhang, Qi Han, Xuhui Li, Yueying Wu, Dajian Zhang, Jianhua Wang, Ran Xu, Li Li, Lianjun Sun

**Affiliations:** 1 State Key Laboratory of Agrobiotechnology, and College of Agronomy and Biotechnology, China Agricultural University, Beijing, 100193 China; 2 Beijing Key Laboratory for Crop Genetic Improvement, College of Agronomy and Biotechnology, China Agricultural University, Beijing, 100193 China; 3 Crop Research Institute, Shandong Academy of Agricultural Sciences, Jinan, 250131 Shandong China; 4 Institute of Bioengineering, Guangdong Academy of Sciences, Guangzhou, 510316 China; 5 College of Agronomy, Shandong Agricultural University, Tai’an, 271018 Shandong China

**Keywords:** QTL mapping, Diversity, Protein content, Oil content, Protein plus oil content, 100-seed weight

## Abstract

As soybean plays an indispensable role in the supply of vegetable oil and protein, balancing the relationship between seed quality and yield traits according to human demand has become an important breeding goal for soybean improvement. Here, 256 intraspecific recombinant inbred lines (RILs), derived from a cross between Qi Huang No.34 (QH34) and Ji Dou No.17 (JD17), were used for quantitative trait loci (QTLs) mapping with remarkable four chemical and physical properties with a purpose for exploring the distribution of excellent alleles in germplasm resources in China. A total of 25 QTLs were detected, of which 10 QTLs inherited the alleles from the parent QH34. Pedigree research on favorable alleles on these QTLs showed the process of excellent alleles pyramided into QH34. Meta-analysis of the 25 QTLs by comparing with existed QTLs in previous study identified 17 novel QTLs. QTLs with pleiotropic effects have been detected. Furthermore, three representative elite recombinant inbred lines in different locations that have great potential in soybean breeding were selected, and finally, four seed weight-related candidate genes were identified. The discovery of these QTLs provides a new guidance for combining the diversity and rarity of germplasm resources, which can effectively increase population genetic diversity and broaden genetic basis of varieties.

**Supplementary Information:**

The online version contains supplementary material available at 10.1007/s11032-021-01211-6.

## Introduction

Soybean [*Glycine max* (L.) Merr.] is an important food and feed crop, and a major source of feed protein and vegetable oil, which is owning to its chemical and physical properties of seed protein, oil content, and 100-seed weight (Yang et al. 2019). Approximately, the protein and oil contents in dry soybean seeds are 40% and 20%, respectively (Rajcan et al. 2005). Seed weight is a component of soybean yield and is an indicator of seed size and seed plumpness. These are complex quantitative genetic traits that are controlled by multiple genes and affected by environmental conditions.

Many studies that focused on QTL mapping to identify QTLs for seed protein content, oil content, seed protein plus oil content, and 100-seed weight in soybean (Chapman et al. 2003; Diers et al. 1992; Orf et al. 1999; Pathan et al. 2013; Sebolt et al. 2000; Li et al. 2018; Yan et al. 2017). To date, 248 QTLs for protein content, 327 for soybean oil content, five for protein plus oil content, and 304 for soybean 100-seed weight distributed across 20 pairs of chromosomes have been reported in SoyBase (http://www.soybase.org). Although numerous seed quality and yield-related QTLs have been detected, only two genes have been cloned, *GmOLEO1* enhanced oil accumulation by affecting triacylglycerol metabolism (Zhang et al. 2019). *GmSWEET39* is positively correlated with soybean seed oil content in terms of the expression level (Miao et al. 2020). Previous studies have shown that soybean seed oil content and 100-seed weight are positively correlated; however, seed protein content is negatively correlated with seed oil content and 100-seed weight, respectively (Wilcox and Zhang 1997; Wilcox 1998). These negative correlations make it difficult to improve those traits simultaneously by traditional breeding.

**Fig. 1:**
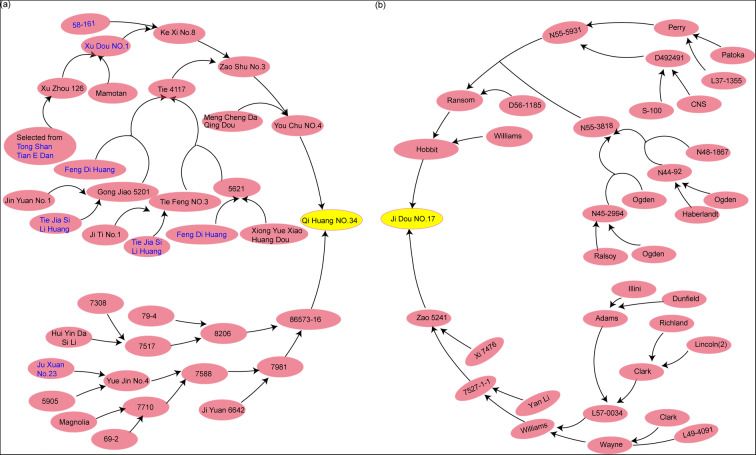
Pedigree analysis of QH34 and JD17. **a**, **b** Pedigree diagram of QH34 and JD17, respectively. QH34 was genetically inherited the predominant alleles from the six elite accessions among the 26 germplasm, which were sequenced by Liu et al. (2020) and highlighted in blue

The rapid progress of soybean pan-genome research has made it possible to obtain pan-genetic sites that control classic traits such as seed protein content, oil content, seed protein plus oil content, and 100-seed weight. China, where cultivar soybean began, has the most abundant soybean germplasm resources and gene pool in the world. Liu et al. (2020) selected 26 accessions (20 of them originated from China), including Qi Huang No.34 and Ji Dou No.17, for de novo assembly to construct a well representative pan-genome, which is representative in terms of phylogenetic relationships and geographic distributions. A pedigree study revealed that QH34 inherited lineages from six out of these 26 accessions in China and the pedigree of JD17 comes from the USA, Japan, North Korea, and other places (Fig. 1). Besides different geographical origins and genetic backgrounds, QH34 and JD17 differ greatly in protein content, oil content, and 100-seed weight, which lead to a relatively wide range of distribution for their progenies; thereby, it is easier to select new varieties with elite traits with different environmental adaptability than improving or cultivating the parental lines.

In this study, QTLs associated with soybean seed protein content, oil content, seed protein plus oil content, and 100-seed weight were detected using the RILs derived from a cross between QH34 and JD17. Pedigree research on favorable alleles on 10 QTLs which inherited the alleles from the parent QH34 showed the process of excellent alleles pyramided into QH34. Four candidate genes related to 100-seed weight were detected as the significantly differential expression between the two parents. The novel QTLs may provide new breeding ideas for improving the seed quality and yield, and guide soybean production and industrialization development.

## Materials and methods

### Plant materials and growth conditions

A population consisted of 256 F_8:9_ RILs was deployed for QTL mapping. The maternal line of the population, QH34, was derived from a cross between Youchu No.4 and 86573-16, and was developed by Shandong Academy of Agricultural Sciences (Institute of Crop Research). The other paternal line, JD17, was derived from a cross between Hobbit and Zao 5241. JD17 was released by Hebei Academy of Agricultural and Forestry Sciences (Institute of Grain and Oil Crops Research) in China.

The two parents and RILs were planted at three locations including Sanya (18.24 N, 109.50′ E), Nanjing (32.04 N, 118.76′ E), and Beijing (39.9 N, 116.24′ E) in 2019, with a row space of 0.5 m (ten seeds in a row) and 0.1 m between lines. The harvested seeds were used for trait evaluation.

### Phenotype evaluation

The harvested seeds of two parents and RILs in Sanya, Nanjing, and Beijing were used for 100-seed weight measurement with an electronic scale (JY3002, Shanghai Sunny Hengping Scientific Instrument Co., Ltd.); the seed oil and seed protein content was analyzed on a Fourier transform near-infrared spectroscopy in Chinese Academy of Agricultural Sciences. All the experiments were performed with three replicates. Seed protein plus oil content was defined as the sum of protein content and oil content.

### Constructing the genetic map

The parental lines, QH34 and JD17, as well as the 256 F_6:7_ RILs population, were sequenced with the reduced representation sequencing method of specific-locus amplified fragment sequencing (SLAF-seq) on an Illumina sequencing platform for developing SNP markers. Sequenced results were aligned to the Williams 82 reference genome (Wm82.a2.v1). A total of 2,223,219 SNPs were obtained in this project, and 1,276,728 SNPs were successfully typed. SNP filter and quality check yielded 6402 high confidence SNP markers that distributed over 20 linkage groups. The 6402 SNP markers were then used for constructing the genetic map using the HighMap software. The total length of the assembled genetic map was 1726.03 cM.

### RNA-seq analysis

The parental lines, QH34 and JD17, were grown in Beijing in 2018. Self-pollinated and premature or mature seeds at stage I (E2 stage), stage II (M2 stage), and stage III (L1 stage) were collected, respectively, and the total RNA was extracted from the seeds tissues with a DP411 RNAprep pure Plant Kit TIANGEN. RNA samples with high quality and RIN (RNA intact number) values (RIN > 6) were used for sequencing on an Illumina platform with the parameter of pair-end 150 (PE150) (Annoroad Gene Technology (Beijing) Co., LTD, Hangzhou, China). Gene expression level was quantified with FPKM (Fragments per Kilobase per Million Mapped Fragments) as below formula:

$$ \mathrm{FPKM}=\frac{10^3\times F}{NL/{10}^6} $$

If FPKM (*A*) is the expression level of gene *A*, then *F* is the number of fragments uniquely aligned to gene *A*, *N* is the total number of fragments uniquely aligned to the reference gene, and *L* is the length of the exon region of gene *A*.

The |log_2_ Fold change|≥1 with *q*-value (adjusted *p*-value) <0.05 was used for differential expressed gene analysis.

### Statistical analysis

The mean value of three replicates for protein, oil, protein plus oil content, and 100-seed weight was used for correlation analysis and QTL mapping. Linear regression analysis (pearson correlation coefficient) of these four traits was analyzed with R statistical package and used to calculate the phenotypic correlation coefficients. Genetic linkage map of QH34 × JD17 was constructed using the *F*_6:7_ population with SNP markers generated from SLAF-seq technology. QTL mapping was conducted with the software QTL IciMapping V4.1 using the inclusive composite interval mapping (ICIM) model (Li et al. 2007). A LOD score > 2.0 was used to declare the existence of a QTL in a specific genomic region.

## Results

### Phenotypic analysis of quality and yield traits in Parents and RILs

**Fig. 2:**
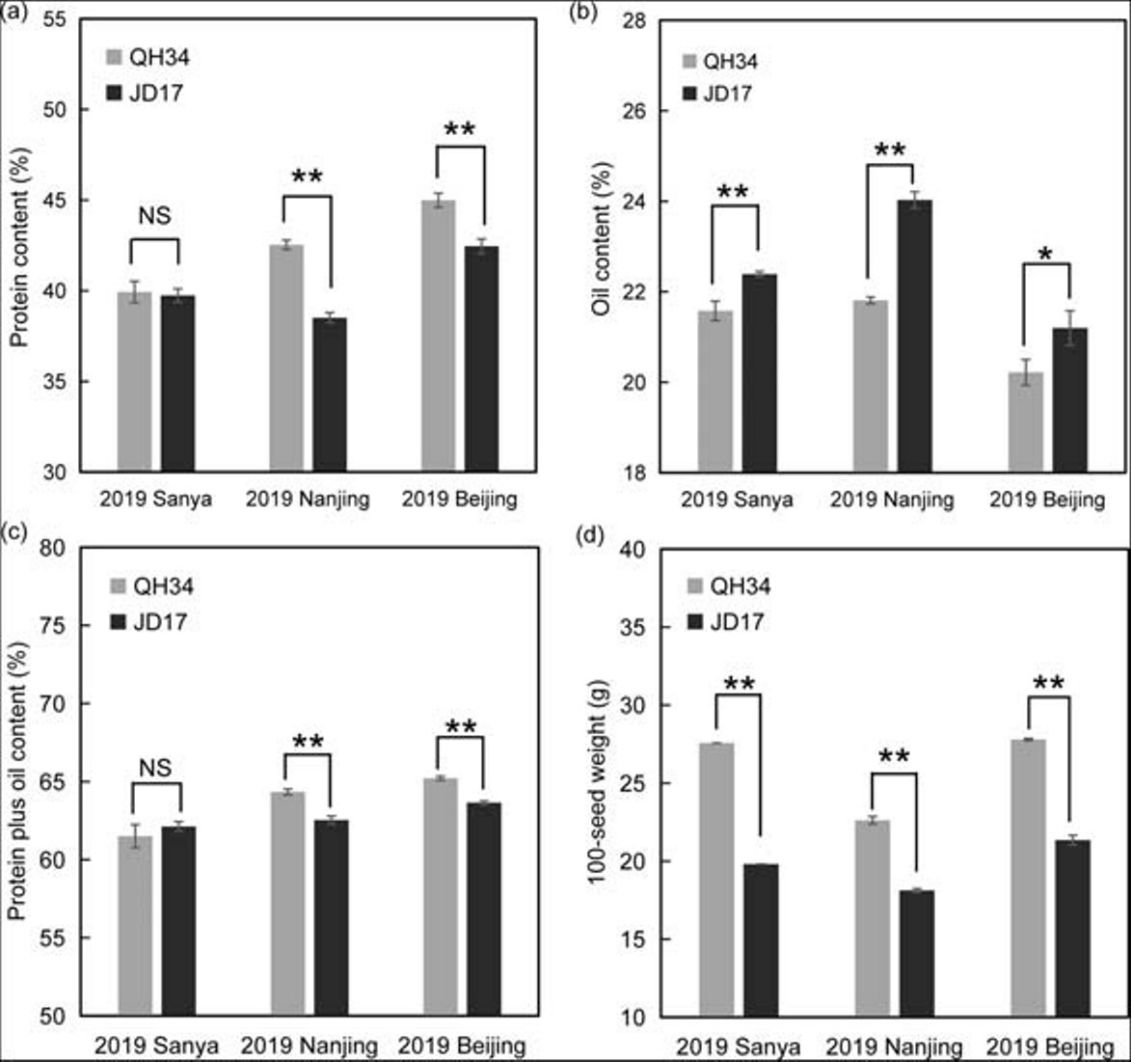
Phenotypes of QH34 and JD17 planted in Sanya, Nanjing and Beijing. **a** The protein content of QH34 and JD17 planted in Sanya, Nanjing, and Beijing. **b** The oil content of QH34 and JD17 planted in Sanya, Nanjing, and Beijing. **c** The protein plus oil content of QH34 and JD17 planted in Sanya, Nanjing, and Beijing. **d** The 100-seed weight planted in Sanya, Nanjing, and Beijing

Seed protein content, seed oil content, seed protein plus oil content and 100-seed weight measured in this study were selected to represent soybean quality and yield traits that differ between QH34 and JD17. QH34 is a typical high-protein inbred line with high yield; the protein content shows great variance planting from south to north of China, which is 39.93%, 42.53%, 44.99% respectively in Sanya, Nanjing, and Beijing. The oil content of QH34 is 21.58%, 21.81%, and 20.22% respectively from south to north; seed protein plus oil content shows an increase from south to north, which is 61.51%, 64.34%, and 65.21% respectively. And the 100-seed weight shows obvious decrease when grown in Nanjing (22.62 g); no significant difference in Sanya and Beijing, 27.57 g and 27.80 g, respectively (Fig. 2). While in JD17, a typical high-oil inbred, the yield is lower than QH34. The protein content of JD17 is 39.74%, 38.51%, and 42.45% respectively from south to north, and the protein content is significantly lower than QH34 in Nanjing and Beijing (*P* < 0.01), while no significant difference when grown in Sanya (Fig. 2a). The oil content shows a highest level of 24.03% in Nanjing, then Sanya and Beijing (22.39% and 21.20%, respectively); Except in Beijing, the oil content of QH34 and JD17 shows extremely significant difference in both Sanya and Nanjing (*P* < 0.01) (Fig. 2b). The seed protein plus oil content of JD17 shows a similar increase from south to north, which is 62.13%, 62.54%, and 63.65%, respectively, in above three locations, and the protein plus oil content is significantly lower than the QH34 in Nanjing and Beijing (Fig. 2c). In terms of 100-seed weight, similar with QH34, JD17 shows a highest level in Beijing (21.35 g) then Sanya (19.82 g) and Nanjing (18.13 g), while it is significantly lower than that was in QH34 (Fig. 2d).

**Fig. 3:**
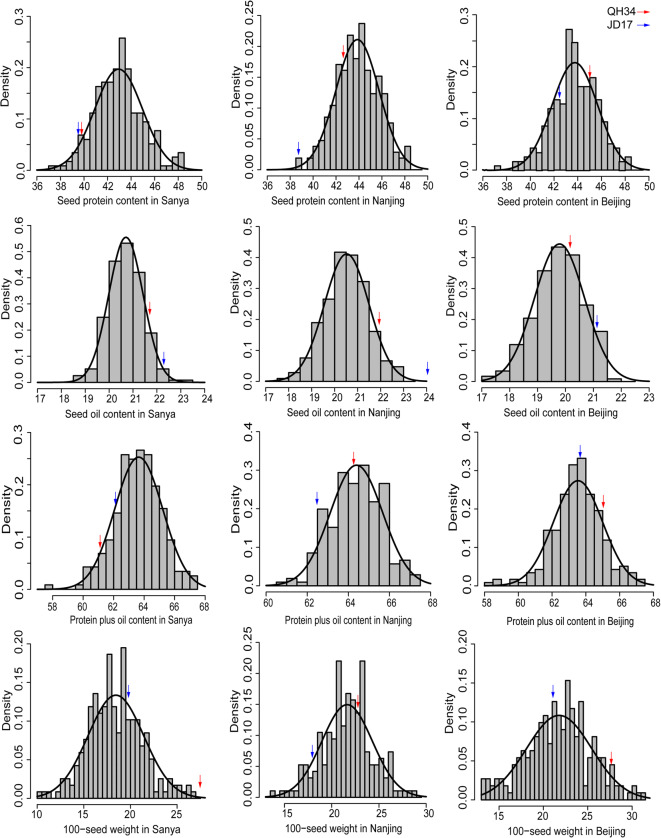
Density plots of seed quality and yield-related traits for RIL populations in three different locations

The phenotypic variation of the RILs at three different locations involving three soybean seed quality and one yield-related traits was documented in Table 1. All four soybean seed–related traits showed continuous variation and approximately in accordance with a normal distribution, with absolute vales of both skewness and kurtosis being less than 1.0, with the exception of kurtosis for 100-seed weight in Sanya (SHW) as well as protein plus oil content and 100-seed weight in Beijing (BPplusO and BHW) (Table 1). Notably, the phenotypic data of all four traits exhibited obvious bidirectional transgressive segregation in this population, indicating the polygenic control of the soybean seed quality and yield traits (Fig. 3). The Pearson correlation analysis for phenotypic values of the RIL population in each location was shown in Table 2. It is obvious that the 100-seed weight is positively correlated with soybean protein content and soybean protein plus oil content, with a significance of *P* < 0.001 in Nanjing and Beijing, and *P* < 0.05 in Sanya. There is no significant (*P* > 0.05) correlation with between 100-seed weight and seed oil content. Seed protein plus oil content has a very significant (*P* < 0.001) positive correlation with the protein content, and a very significant (*P* < 0.001) negative correlation with the oil content in each location. An extremely high negative correlation existed between seed protein content and seed oil content in each location (*P* < 0.001) (Table 2).

**Table 1:** Seed quality and yield-related trait performances of RIL population (QH34 × JD17) in three locations from north to south China

Trait name^a^	Mean^b^	SD	Skewness	Kurtosis	Range	*W*-test^c^	*P*-value^d^
SP	42.9415	2.0238	0.202	0.2002	37.29–48.50	0.9818	0.4005
SO	20.7027	0.7188	0.1408	0.3489	18.67–23.33	0.9924	0.9849
SPplusO	63.6443	1.5776	−0.1339	0.6352	57.67–67.92	0.9901	0.9412
SHW	18.492	3.0942	0.5265	1.1439	10.38–31.20	0.9805	0.3029
NP	43.8713	1.8908	−0.0732	−0.2293	38.58–48.21	0.9809	0.3673
NO	20.524	0.9694	0.096	0.2206	17.70–23.56	0.9895	0.9229
NPplusO	64.3953	1.2745	0.006	−0.1106	60.77–67.89	0.9864	0.7725
NHW	21.6327	2.6678	−0.0973	0.1465	13.63–28.53	0.985	0.6902
BP	43.7492	1.9164	−0.2159	0.1473	37.28–48.47	0.9861	0.7361
BO	19.7866	0.8984	−0.1869	0.0698	17.01–22.56	0.9894	0.9195
BHW	21.5184	4.1125	−0.6288	1.3539	6.07-31.46	0.9687	0.006115
BPplusO	63.5357	1.4578	−0.411	1.1571	58.08–67.08	0.9753	0.0706

^a^Trait names SP, SO, SPplusO, and SHW represent seed protein content in Sanya, seed oil content in Sanya, seed protein plus oil content in Sany and 100-seed weight in Sanya, respectively

NP, NO, NPplusO, and NHW represent seed protein content in Nanjing, seed oil content in Nanjing, seed protein plus oil content in Nanjing, and 100-seed weight in Nanjing, respectively. Content in Beijing, seed protein plus oil content in Beijing, and 100-seed weight in Beijing, respectively

^b^Mean of the phenotypic trait

^c^The Shapiro Wilk *W*-statistic for the test of normality

^d^*P*-value of the *W*-test of normality

BP, BO, BPplusO and BHW represent seed protein content in Beijing, seed oil

**Table 2:** Phenotypic correlations (upper right diagonal) among protein content, oil content, protein plus oil content, and 100-seed weight in 256 recombinant inbred lines in Sanya (S), Nanjing (N), and Beijing (B)

Sanya traits	SP	SO	SPplusO	SHW
SP	1			
SO	−0.74***	1		
SPplusO	0.95***	−0.49***	1	
SHW	0.18*	−0.12	0.18*	1
Nanjing traits	NP	NO	NPplusO	NHW
NP	1			
NO	−0.79***	1		
NPplusO	0.88***	−0.41***	1	
NHW	0.35***	−0.04	0.5***	1
Beijing traits	BP	BO	BPplusO	BHW
BP	1			
BO	−0.7***	1		
BPplusO	0.9***	−0.32***	1	
BHW	0.31***	0.05	0.45***	1

^*^Significant at the 0.05 probability level

^**^Significant at the 0.01 probability level

^***^Significant at the 0.001 probability level

### QTL mapping of seed quality and yield traits in the RIL population

**Fig. 4:**
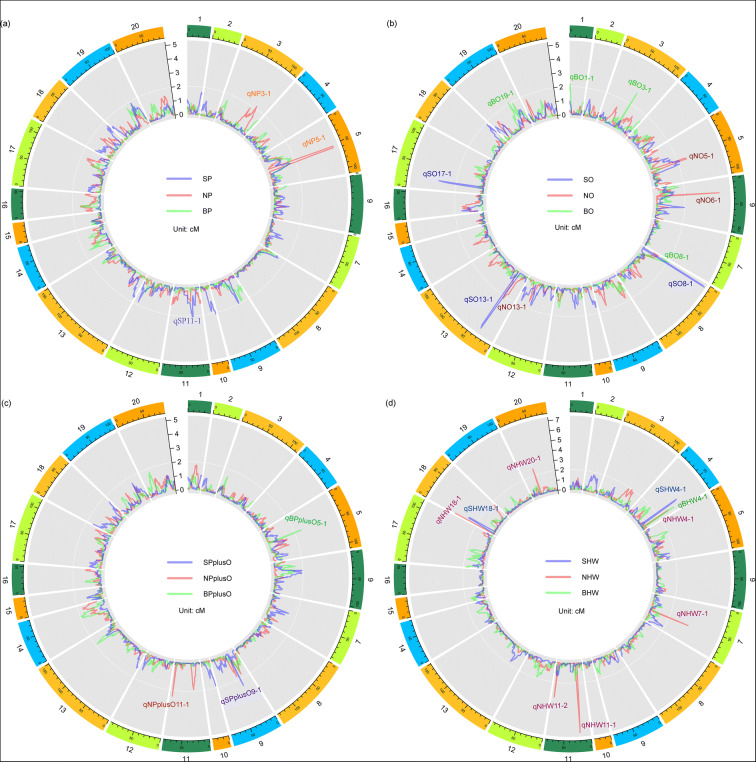
QTL distribution for seed quality and yield-related trait detected from the RIL population across three different locations. **a** The QTL distribution for seed protein content. **b** The QTL distribution for seed oil content. **c** The QTL distribution for seed protein plus oil content. **d** The QTL distribution for 100-seed weight. Different colored lines within the chromosome cycle represent the chromosome positions and LOD values for QTLs detected for protein content in Sanya (SP), protein content in Nanjing (NP), and protein content in Beijing (BP); oil content in Sanya (SO), oil content in Nanjing (NO), and oil content in Beijing (BO); protein plus oil content in Sanya (SPplusO), protein plus oil content in Nanjing (NPplusO), and protein plus oil content in Beijing (BPplusO); 100-seed in Sanya (SHW), 100-seed weight in Nanjing (NHW), and 100-seed weight in Beijing (BHW). Detailed QTL information is listed in Table 3

A total of 25 significant QTLs across 14 chromosomes (including chromosomes 1, 3, 4, 5, 6, 7, 8, 9, 11, 13, 17, 18, 19, and 20) were identified with the explained phenotypic variance of 3.07–11.24% and the LOD values of 2.07–6.93 (Table 3; Fig. 4). These 25 QTLs corresponded to the four soybean seed quality and yield traits in different locations, with three for protein content, ten for oil content, three for seed protein plus oil content, and nine for 100-seed weight. Among that, Chr 11 contains most QTLs involving 100-seed weight, protein plus oil content in Nanjing, and protein content in Sanya. Chr 4 contains three independent QTLs involving 100-seed weight in three locations. Chr 5 also contains three tandem QTLs involving oil and protein content in Nanjing, and protein plus oil content in Beijing (Table 3). The highest explained phenotypic variance and LOD value were found for a 100-seed weight QTL (*qNHW11-1*). Ten QTLs with a negative additive effect inherited the contributable alleles from the parent QH34, while 15 with a positive additive effect inherited the contributable alleles from the parent JD17 (Table 3).

**Table 3:** Summaries of the QTLs identified in Sanya, Nanjing, and Beijing

Traits^a^	QTLs^b^	Chromosome	Left marker^c^	Right marker^d^	LOD	PVE (%)	Add	Left CI	Right CI	Reported
SP	*qSP11-1*	11	M11C28321565	M11C29946706	2.0753	3.341	−0.3934	22.5	26.5	Novel
NP	*qNP3-1*	3	M3C5929028	M03C6367422	2.1664	3.0745	−0.371	87.5	95.5	*Seed protein 36-36, cqSeed protein-004*
	*qNP5-1*	5	M5C31683037	M5C32119045	4.882	7.4966	0.5842	52.5	58.5	Novel
SO	*qSO8-1*	8	M8C46265715	M8C46466790	5.1606	7.4433	0.2173	5.5	7.5	Novel
	*qSO13-1*	13	M13C39648566	M13C40855202	4.8215	6.9474	0.21	30.5	34.5	Novel
	*qSO17-1*	17	M17C38410454	M17C38470629	3.2334	4.4005	0.1669	17.5	21.5	Novel
NO	*qNO5-1*	5	M5C31683037	M5C32119045	2.6628	5.6047	−0.2232	52.5	59.5	Novel
	*qNO6-1*	6	M6C41529927	M6C42101650	4.4589	9.3784	−0.2869	27.5	28.5	Seed oil 5-3
	*qNO13-1*	13	M13C40855233	M13C41113159	2.1872	4.2524	0.1927	23.5	32.5	Novel
BO	*qBO1-1*	1	M1C55100231	M1 C55269760	2.1616	3.3801	0.1722	0	3.5	Novel
	*qBO3-1*	3	M3C34758945	M3C34782039	2.8894	4.8979	0.2073	65.5	66.5	Novel
	*qBO8-1*	8	M8C47411750	M8C47686515	2.1099	3.3175	0.1707	0	5.5	Novel
	*qBO19-1*	19	M19C35829392	M19C36028657	2.0946	3.2722	0.17	65.5	70.5	*Seed oil 2-7, Seed oil 5-3*
SPplusO	*qSPplusO9-1*	9	M9C42057126	M9C42180462	2.5231	4.2209	0.3281	25.5	26.5	Novel
NPplusO	*qNPplusO11-1*	11	M11C2375493	M11C2638141	2.4243	6.2788	−0.296	77.5	78.5	Novel
BPplusO	*qBPplusO5-1*	5	M5C33573129	M5C33653958	2.5077	4.6412	0.3148	45.5	46.5	Novel
SHW	*qSHW4-1*	4	M4C6282642	M4C6294438	4.4304	5.4732	−0.8551	59.5	62.5	*Seed weight 54-1, Seed weight 54-2, Seed weight 38-2*
	*qSHW18-1*	18	M18C54440397	M18C56700579	2.9666	3.5919	0.6974	4.5	8.5	*Seed weight 3-3*
NHW	*qNHW4-1*	4	M4C4011194	M4C4018279	3.1256	5.0014	−0.5697	70.5	71.5	*Seed weight 38-2*
	*qNHW7-1*	7	M7 C7735328	M7 C8444155	4.0805	6.3256	0.6406	55.5	56.5	Novel
	*qNHW11-1*	11	M11C30705855	M11C31732545	6.9396	11.2408	−0.8621	17.5	21.5	*Seed weight 35-9*
	*qNHW11-2*	11	M11C2375493	M11C2638141	3.4888	5.459	−0.5963	77.5	78.5	Novel
	*qNHW18-1*	18	M18C57529669	M18C57583908	4.3701	6.7846	0.6672	0	0.5	Novel
	*qNHW20-1*	20	M20C39865792	M20C39942933	2.6691	4.1296	0.5172	30.5	31.5	*Seed weight 15-5, Seed weight 36-5*
BHW	*qBHW4-1*	4	M4C 3637603	M4C3722802	3.7459	7.4372	−1.1137	71.5	72.5	Novel

^a^Trait names SP, SO, SPplusO, and SHW represent seed protein content in Sanya, seed oil content in Sanya, seed protein plus oil content in Sanya, and 100-seed weight in Sanya, respectively

NP, NO, NPplusO, and NHW represent seed protein content in Nanjing, seed oil content in Nanjing, seed protein plus oil content in Nanjing, and 100-seed weight in Nanjing, respectively

BP, BO, BPplusO, and BHW represent seed protein content in Beijing , seed oil content in Beijing, seed protein plus oil content in Beijing, and 100-seed weight in Beijing, respectively

^b^QTLs were named following “q” + trait + chromosome + its order in this chromosome

^c,d^Markers were named following marker (M) + chromosome number + “C” + its physical position in Wm82.a2.v1

### QTLs for protein content

In this study, a total of three QTLs related to seed protein content were identified with the explained phenotypic variance of 3.07–7.50% in two locations at Sanya (*qSP11-1*) and Nanjing (*qNP5-1* and *qNP3-1*). QTL *qNP5-1* (31.6–32.1 Mb) has the highest LOD value of 4.88 and explained 7.50% of the phenotypic variation. The contributable alleles for the protein content–related two QTLs (*qNP3-1* and *qSP11-1*) from this study were inherited from the high-protein parent QH34, while the contribution of *qNP5-1* was inherited from the low protein parent JD17 (Table 3; Fig. 4a). No confidential QTL-controlling soybean seed protein content was detected in Beijing.

### QTLs for oil content

Compared to the protein content, oil content is relatively stable and insensitive to the environmental change. Here, totally ten soybean seed oil content–related QTLs were identified in Sanya (*qSO8-1*, *qSO13-1*, and *qSO17-1*), Nanjing (*qNO5-1*, *qNO6-1*, and *qNO13-1*), and Beijing (*qBO1-1*, *qBO3-1*, *qBO8-1*, and *qBO19-1*), which explained phenotypic variation of 3.27–9.38%. All three oil content–related QTLs detected in Sanya and four detected in Beijing were inherited from the high-oil parent JD17, except two QTLs (*qNO5-1* and *qNO6-1*) identified in Nanjing, which were inherited from the low oil parent QH34. More interestingly, *qNO6-1* explained the highest phenotypic variance of 9.38%, with the LOD value of 4.46. *qSO8-1* on Chr 8 (46.3–46.5 Mb) has the highest LOD value of 5.16 and explained 7.44% of the phenotypic variation (Table 3; Fig. 4b).

### QTLs for protein plus oil content

For the protein plus oil content trait, QTLs were detected in each of Sanya (*qSPplusO9-1*), Nanjing (*qNPplusO11-1*), and Beijing (*qBPplusO5-1*) with the LOD value around 2.5, which explained phenotypic variance from 4.22–6.28%. QTLs detected in both Sanya and Beijing were inherited from the parent JD17, while the QTL on Chr 11 detected in Nanjing was inherited from the parent QH34. The QTL *qNPplusO11-1* on Chr 11 (2.37–2.63 Mb) detected in Nanjing explained the highest phenotype variation of 6.27%, and the contributable allele was inherited from large-high-protein seed parent QH34, indicating that the protein plus oil content is mostly related to the seed protein content and 100-seed weight. Interestingly, the other two QTLs of *qSPplusO9-1* (Chr 9:42.1–42.2 Mb) and *qBPplusO5-1* (Chr 5:33.57–33.65 Mb) detected in Sanya and Beijing, respectively, were inherited from the small-high-oil seed parent JD17 (Table 3; Fig. 4c).

### QTLs for 100-seed weight

Nine QTLs related to 100-seed weight of soybean were identified, including two QTLs (*qSHW4-1* and *qSHW18-1*) detected in Sanya, six QTLs (*qNHW4-1*, *qNHW7-1*, *qNHW11-1*, *qNHW11-2*, *qNHW18-1*, and *qNHW20-1*) detected in Nanjing and one QTL (*qBHW4-1*) detected in Beijing. QTL *qNHW11-1* explained the highest phenotypic variance of 11.24% to 100-seed weight when grown in Nanjing, followed by *qBHW4-1*, explained a relatively high phenotypic variance of 7.44% to 100-seed weight when grown in Beijing. The contribution of most of the 100-seed weight-related QTLs were inherited from the high weight parent QH34 while only four QTLs (*qNHW18-1*, *qNHW20-1*, *qNHW7-1*, and *qSHW18-1*) were inherited from the low weight parent JD17 (Table 3; Fig. 4d).

### Pedigree research on QTL favorable alleles

QH34 is a representative decent variety in China with wide adaptability and high yield and quality, which is proved by QH34-derived lineages from six representative accessions: 58–161, Xu Dou No.1, Tong Shan Tian E Dan, Feng Di Huang, Tie Jia Si Li Huang, and Ju xuan No.23. A pedigree research showed that six accessions above had different ways to contribute to the QTL favorable alleles on the 10 QTL-inherited alleles from the parent QH34.

**Fig. 5:**
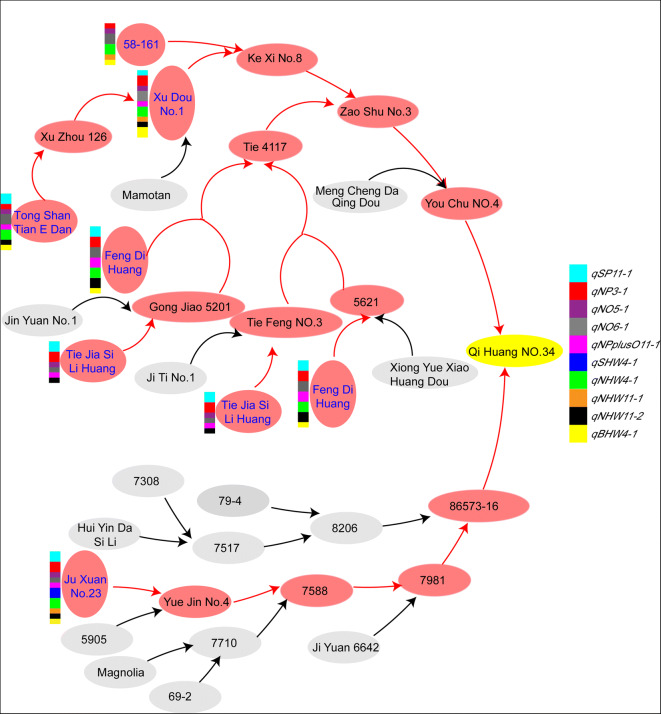
Pedigree research on QTL favorable alleles inherit from QH34. The alleles contributed by QH34 on different QTLs in six accessions are denoted as colored square. The accession contributed whole QTL favorable alleles and half of the alleles are denoted by square and half a square (rectangle), respectively. The red ellipse and arrow means the path of the QTL favorable alleles pyramided into QH34, and the gray ellipse means the unstudied accessions

Some alleles on QTLs were contributed by a single accession. For example, the alleles on *qSHW4-1*, increased 100-seed weight, were derived only from Ju Xuan No.23, and the alleles were not existed in other five accessions. Some accessions can provide complete alleles on QTL, for example, the alleles on *qNHW4-1* were provided by 58–161, Xu Dou No.1, Tong Shan Tian E Dan, Feng Di Huang, and Ju xuan No.23 (any of them can provide complete *qNHW4-1* favorable alleles individually). For another example, Xu Dou No.1 contained the whole favorable alleles on *qBHW4-1*. Some QTL favorable alleles were provided by different accessions, for example, the alleles on *qNHW11-1* partly derived from 58 to 161 and Xu Dou No.1, and partly derived from Ju Xuan No.23 (Table S1; Fig. 5).

The alleles on QTLs that exist or partially exist in multiple accessions occurred alleles pyramiding, for example, the QTL favorable alleles in 58–161 and Xu Dou No.1 pyramided in Ke Xi No.8, and QTL favorable alleles in both Tie Jia Si Li Huang and Feng Di Huang pyramided in Tie 4117. With the continuous hybridization, the QTL favorable alleles pyramided again until they pyramided into QH34. QTL favorable alleles pyramiding may make it difficult to lose the excellent genetic sites that affected the development of important plant traits. Interestingly enough, QTL favorable alleles on *qSP11-1* were derived from Tong Shan Tian E Dan, but were partly from Xu Dou No.1, indicating that the QTL favorable alleles would be partially lost during the gene transfer process (Fig. 5).

## Discussion

### Identification of novel and pleiotropic QTLs

**Fig. 6:**
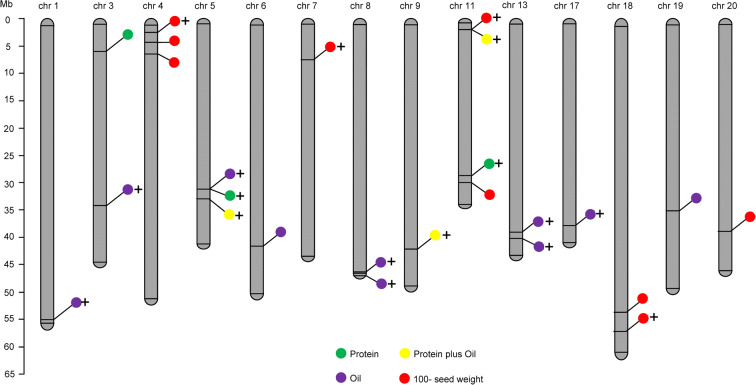
Meta-QTLs on physical map as detected in QH34 × JD17. The identified QTLs were denoted as colored dots. Novel QTLs identified in this study were marked with a plus (+)

SoyBase (http://www.soybase.org) is the soybean database that includes all the QTLs or genes that have been identified so far. We further compared our QTL mapping results with the related QTLs in soybase. A total of eight QTLs shared the same or overlapping confidence intervals with the QTLs identified in previous studies, including one for seed protein content (Mao et al. 2013; Pathan et al. 2013), two for seed oil content (Diers et al. 1992; Lee et al. 1996), and five for 100-seed weight (Hacisalihoglu et al. 2018; Han et al. 2012; Hyten et al. 2004; Mian et al. 1996; Yang et al. 2011). More importantly, we found 17 novel QTLs controlling seed quality and yield-related traits (Fig. 6; Table 3).

QTLs with pleiotropic effects for seed protein content and oil content have been reported in previous studies (Brummer et al. 1997; Hyten et al. 2004; Mansur et al. 1996; Orf et al. 1999; Sebolt et al. 2000). Most of the studies showed that the oil and protein paradoxically existed in soybean, thus it is reasonable that one QTL has a positive effect on oil or protein while having a negative effect on the other (Wilcox 1998). However, there are few studies of pleiotropic effects on both seed weight and seed protein plus oil content. In this study, one QTL located on Chr 5 between markers M5C31683037 and M5C32119045 identified in Nanjing was associated with both seed protein content (*qNP5-1*) and seed oil content (*qNO5-1*). One QTL located on Chr 11 between markers M11C2375493 and M11C2638141 can regulate both seed protein plus oil content (*qNPplusO11-1*) and 100-seed weight (*qNHW11-2*). These results indicated these QTLs associated with four traits may have pleiotropic effects.

### The relationship between environment and phenotype

The change trend of the 100-seed weight of the parents showed that genotype contributes more to 100-seed weight than environment. But for the same genotype, 100-seed weight will change along with change of the latitude, both QH34 and JD17 have the highest 100-seed weight in Beijing. The variation is not significant in protein content between QH34 and JD17 in Sanya, which demonstrated that the protein content is easier affected by environmental than the genetic factor. The pattern of protein content is similar to the 100-seed weight in JD17, while the protein content experienced a significant increase from south to north in QH34. Both QH34 and JD17 have higher protein content in Beijing than the other two locations. Although QH34 have greater protein content than JD17 in general, it is unwise to grow QH34 in Sanya because there is no significant advantage over JD17 once grow them in Sanya. For both QH34 and JD17, the oil content is highest in Nanjing; it shows an increase and followed by a decrease from south to north, indicating that it is more beneficial for the accumulation of oil in Nanjing. However, the high oil potential of JD17 cannot be released in Beijing. The changes of protein plus oil content were generally consistent with the trend of protein from south to north, indicating the contribution of “protein plus oil content” mainly comes from “protein” rather than “oil,” which is also supported by the fact that the protein content (~40%) takes up more of seed weight than the oil content (~20%), and the seed weight is often relies on the change of protein content.

### Correlation among seed quality and yield traits

A highly significant negative correlation existed between seed protein and oil content in our study, which was in accordance with previous research (Wilcox 1998), indicating that it is difficult to increase both the oil and the protein content in soybean. It has been reported that there are large variations of correlation between seed weight and seed quality (seed protein and oil content), including positive and negative effects (Reinprecht et al. 2006; Specht et al. 2001). Here, the 100-seed weight was positively correlated with soybean protein, while no significant correlation with the oil content. However, some studies proved that soybean yield is negatively correlated with protein content but positively correlated with oil content (Wilcox and Zhang 1997). As we know, protein takes up more weight component than that is oil in soybean seed (~40% vs. 20%); 100-seed weight is only one of the component that contribute to the yield, and it is possible other yield factors also play the roles, i.e., by reducing the grain number per plant, resulting in a decrease of yield in their studied populations. It is worth noting that, among 256 RILs of QH34 × JD17, one RIL family QJ163 contains QTL favorable alleles that simultaneously increased soybean 100 seed weight, oil content, and protein plus oil content in Beijing. Moreover, QJ186 and QJ235 contain QTL favorable alleles that could simultaneously increase seed oil, protein, protein plus oil content, and 100 seed weight as well when grown in Sanya. Those families could be used as the excellent germplasms for soybean breeding.

### Four candidate genes response for a stable QTL related to 100-seed weight

A QTL identified in all three locations was declared to be stable. In this study, only QTLs related to 100-seed weight on Chr 4 were detected in each three locations, and covers 2.65-Mb interval (3.64–6.29 Mb); there are 296 genes in this region. Coincidentally, the growth adaptability–related *J* gene, located around 4.1 Mb (4.07–4.08 Mb) on Chr 4, is a member of 296 genes (Lu et al. 2017). Comparing the published genome data of QH34 and JD17 (Liu et al. 2020), no polymorphism was detected in *J* gene between the two parents, which may explain why the 100-seed weight is not affected by the environmental changes. Pedigree research on the *J* gene in QH34 showed that this haplotype came from 58 to 161, Feng Di Huang and Ju Xuan No.23 (Table S1).

**Fig. 7:**
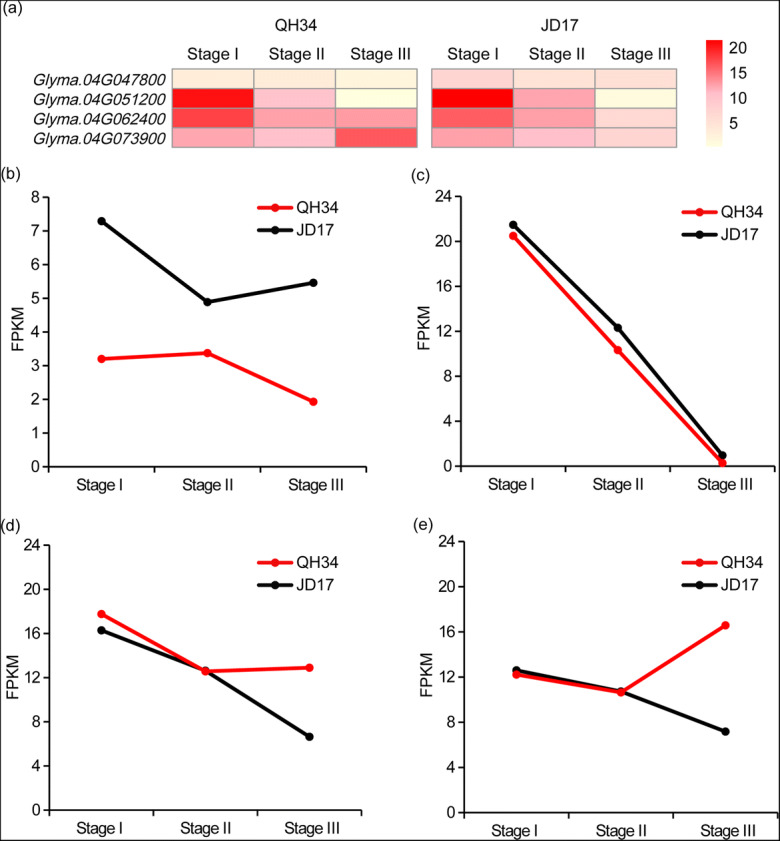
Heat map and gene expression pattern of candidates detected in the common 100-seed weight-related QTL detected in three locations. **a** Heat map of the genes expression detected by RNA-seq analysis (FPKM) during seed development. **b**, **c**, **d**, **e** Gene expression pattern of *Glyma.04G047800*, *Glyma.04G051200*, *Glyma.04G062400*, and *Glyma.04G073900*

To identify the candidate genes that related to the soybean 100-seed weight, RNA-seq analysis of the different developed seeds for two parental lines was performed. According to soybean seed establishment and development, differential expressed genes (DEGs) between two parental lines of QH34 and JD17 were extracted, combine with the QTLs that contributable for 100-seed weight, we discovered that only one DEG was identified in stage I and 23 DEGs were detected in stage III, and no DEG detected in stage II. In previous report, the seed formation is a process of cell division to cell number increasing, and cell elongation to cell volume enlargement (Liu et al. 2007). Here, we focused on DEGs that carry sequence variation (SNP variation or insertion-deletion) in the QTL of interested, combine with gene function annotation, four candidate genes of *Glyma.04G047800*, *Glyma.04G051200*, *Glyma.04G062400*, and *Glyma.04G073900,* involved in the processes of cell development and seed development were identified. The expression pattern of those four genes from stage I to III was totally different, which may explained by involving in different pathways (Fig. 7). *Glyma.04G047800* encodes an APETALA2 (AP2)-like factor that has been reported in *Arabidopsis*, it plays an important role in the regulation of floral organs in *Arabidopsis*, also regulates the seed development (Jofuku et al. 1994). *Glyma.04G051200* is involved in the process of cell differentiation and cell wall modification, and it may contributed to the 100-seed weight by increasing the cell numbers and seed size. *Glyma.04G062400* encodes a chromatin remodeling factor CHD3 (PICKLE), a protein that regulates cytokinin signaling in late processes, of which cytokinin has proved to regulate the seed size by regulating the growth of embryonic cells during seed development (Furuta et al. 2011; Werner et al. 2003; Riefler et al. 2006). *Glyma.04G073900* is a homolog of *AT4G31160,* which encodes a DDB1-CUL4 ASSOCIATED FACTOR1 (DCAF1) protein capable of interacting with DDB1 and associating with CUL4, likely as part of a nuclear E3 ubiquitin ligase complex (Zhang et al. 2008). Previous study has proven that proteins related to the ubiquitin pathway are involved in regulating plant seed size (Li et al. 2008). More molecular and biology experiments were required for further evidence of the functions in terms of above four candidates.

## Implications for breeding

Through pedigree analysis in the condition of the clear genetic background, we can find the origin of QTL favorable alleles, and clearly see the process of the QTL favorable alleles pyramiding into the offspring. In the breeding process, we should achieve the diversity of breeding materials, so that with the pyramiding of excellent alleles, the progenies will become an aggregation of excellent genes.

The QTLs (such as *qNP3-1*, *qSHW4-1*, *qNHW4-1*) identified in previous studies indicate that such excellent alleles widely existed in multiple accessions and they may be retained in the breeding process through phenotypic value selection. We also found QTLs (such as *qSP11-1*, *qNO5-1*, *qNPplusO11-1*) that are undetected in previous studies, indicating that such QTL favorable alleles are unique in the RILs derived from QH34 and JD17 and these types of excellent alleles are relatively rare in the soybean population.

As we all know, there is a bottleneck effect in the domestication process, which causes rare alleles that are more likely to be lost than the widespread alleles, and eventually lead to a smaller gene pool. It is widely known that reduction in genetic diversity may result in the population that is more susceptible to disease. What’s more, in the breeding process, breeders tended to select minority excellent germplasm, which caused the germplasm to be used too frequently to result in the loss of genetic diversity of the varieties in production. The narrow genetic basis of varieties has limited the further improvement of quality and yield, which is essential to maintain the increasing population. Fortunately, the discovery and utilization of these rare alleles can effectively increase population genetic diversity and broaden genetic basis of varieties, so that there would be higher levels of standing genetic variation, making it more likely that some individuals would happen to have a gene variant that conferred the ability to increase quality and yield.

All in all, only by combining the diversity and rarity of germplasm resources can we breed varieties with wide adaptability and high quality.

## Supplementary information

Table S1 (XLSX 10 kb)
